# Distrustful, Dissatisfied, and Conspiratorial: A Latent Profile Analysis of COVID-19 Vaccination Rejection

**DOI:** 10.3390/ijerph191610096

**Published:** 2022-08-15

**Authors:** Monika Lamot, Katja Kerman, Andrej Kirbiš

**Affiliations:** 1 Department of Sociology, Faculty of Arts, University of Maribor, 2000 Maribor, Slovenia; 2 Department of Psychology, Faculty of Arts, University of Maribor, 2000 Maribor, Slovenia

**Keywords:** trust, science, government, conspiracy theories, COVID-19 vaccination, latent profile analysis

## Abstract

Trust in science and scientists, satisfaction with the national government, and endorsement of conspiracy theories are important factors in the decision to be vaccinated. In this study, we investigated whether there are different profiles of individuals depending on the above factors and whether they differ in their sociodemographic composition. We used data from Round 10 of the European Social Survey for Slovenia, employing a nationally representative sample of 1252 participants. Based on latent profile analysis, three distinct profiles emerged: Profile 1 expressed moderate trust in science, satisfaction with government, and high endorsement of conspiracies; Profile 2 expressed low trust and satisfaction and moderate endorsement of conspiracies; Profile 3 expressed high trust and satisfaction and low beliefs in conspiracy theories. In addition, Profile 3 expressed the strongest support for vaccination and Profile 2 the lowest. Our results suggest that distrust, dissatisfaction, and the presence of conspiracy theories are the “perfect storm” for vaccination rejection. In contrast, despite conspiracy theories, a certain level of trust and satisfaction may reduce vaccination rejection.

## 1. Introduction

In the wake of the COVID-19 health crisis, many countries are faced with vaccine hesitancy and low vaccination rates. Slovenia has low COVID-19 vaccination rates compared to other European Union members [1] and was ranked high amongst vaccine-hesitant countries even before the COVID-19 pandemic [2]. Although childhood immunization is mandatory in Slovenia, COVID-19 vaccination is not compulsory for any group [3]. In addition to low vaccination rates, Slovenians also endorsed conspiracy theories [4] and high levels of distrust in government and health institutions during the COVID-19 pandemic [5]. This is particularly problematic since increased trust in institutions [6,7,8,9,10,11,12] and low endorsement of conspiracy theories [13] contribute to higher vaccination rates, highlighting the need to examine different groups of individuals regarding their perception of science, government, and conspiracy beliefs.

### 1.1. Trust in Scientists, Satisfaction with the Government and Endorsement of Conspiracy Theories about COVID-19 Vaccination

Trust in science and scientific experts significantly contributes to positive attitudes toward vaccination [6,8,14,15,16,17]. For example, it was observed that the intention to get vaccinated against COVID-19 is related to trust in scientific research, which decreased during the pandemic [14]. Trust in science is particularly important in times of social change and distress since such circumstances can contribute to the spread of conspiracy theories. Conspiracy beliefs provide internally consistent explanations that permit people to preserve their beliefs in the face of uncertainty or contradiction [18]. In uncertain times, such as the COVID-19 pandemic, conspiracy theories can, therefore, provide explanations of present events that could ease an individual’s distress [19]. However, such explanations are not consistent with scientifically based explanations of the world and current events. Furthermore, it has been shown that conspiracy beliefs predict mistrust in science [20]. Individuals believing in COVID-19 conspiracy theories, in turn, express lower intention for COVID-19 vaccination [7,13].

In addition, it has been shown that (dis)satisfaction with the government dealing with the pandemic also impacts vaccine uptake [21,22]. Furthermore, dissatisfaction with the government increases the endorsement of conspiracy theories about the origins of the COVID-19 virus [23].

### 1.2. Latent Profile Analysis in Researching Vaccine Attitudes

It is important to stress that the proportions of individuals expressing high levels of distrust of vaccines are small at the population level (the so-called “small pocket” problem). Consequently, such subsamples may be overlooked in traditional regression models [24]. Therefore, it is necessary to examine different groups of individuals in terms of vaccine attitudes using latent profile analysis (LPA), especially since the literature indicates that groups of vaccine-hesitant or rejecting individuals may vary in vaccine acceptance. In LPA, an individual or group is identified based upon their configuration of personal and environmental attributes [25].

People grouped into different vaccination hesitancy profiles also tend to differ with regard to sociodemographic and socioeconomic characteristics, although inconsistently across studies. For example, five profiles have been identified among Hong Kong health workers. The analysis revealed that the profile with the highest intention to vaccinate was characterized by lower educational attainment, more long-term illness, higher work stress, and greater trust in the government, among other factors [26]. In another study, motivation to vaccinate against both COVID-19 and seasonal influenza was examined in Taiwan [27]. Individuals with low motivation to vaccinate against both infectious diseases had a high school degree or less, were not employed in health care, and were less likely to have received information about COVID-19 vaccination from friends, family, traditional media, and the Internet [27]. The latter is particularly interesting since obtaining information regarding vaccines from the Internet has previously been identified as a predictor of vaccine hesitancy [28,29,30]. In Spain, it was found that the profile of those who expressed the most reservations about COVID-19 vaccination included highly educated individuals who expressed high levels of liberal attitudes while expressing reservations about genetically modified organisms (GMOs) and nuclear power. This group also reported getting most of their information/news on science issues through the Internet. The second most hesitant group consisted of younger individuals who were less educated and more on the politically extreme—both on the left and right [24]. These studies point to the complex nature of attitudes toward vaccination and the heterogeneity of groups expressing them. Our study adds to the existing literature by examining the role of trust in scientists, satisfaction with government, and endorsement of conspiracies, while at the same time, exploring sociodemographic differences.

The aim of the present study is to investigate whether there are different profiles of individuals in terms of their attitudes toward satisfaction with government, trust in scientists, and belief in conspiracy theories, with these variables being latent indicators. At the same time, we were interested in whether the latent profiles differed in their sociodemographic composition and attitudes toward vaccination against COVID-19. Specifically, the study addressed three research questions:

- RQ 1: Are there different latent profiles of individuals that quantitatively and qualitatively differ in terms of their trust in scientists, their satisfaction with the national government, and their belief in conspiracy theories?
- RQ 2: Do latent profiles differ by sociodemographic composition (gender, age, education, and political orientation)?
- RQ 3: Do the identified profiles differ in their attitudes toward vaccination against COVID-19?

## 2. Materials and Methods

### 2.1. Participants

We used data from the Round 10 European Social Survey (ESS) for Slovenia [31]. ESS sampling is representative of all persons 15 years of age and older, and individuals are selected at each stage using strict random probability methods. For Slovenia, the Central Register of the population was used as the sampling frame. The data were collected using a two-stage probability sampling procedure, with stratification at the first stage. The data were collected between 18 September 2020 and 26 August 2021 [31]. The nationally representative sample comprises 1252 participants, with an average age of 49.4 years. The sample is evenly represented in terms of gender (47.2% men, 52.8% women). Most participants have a high school diploma (53.5%), followed by BA or similar degree (25.3%). The sample predominantly expresses distrust of the Slovenian parliament (M = 3.86), politicians (M = 2.72), and political parties (M = 2.78). Descriptive statistics and correlations between variables of interest can be found in Supplementary Materials.

### 2.2. Materials

#### 2.2.1. Latent Indicators

Trust in scientists was measured with an 11-point scale (0 = no trust at all; 10 = complete trust), with the question, “How much do you trust scientists?”. Satisfaction with the government was tapped with a question, “Now thinking about the Slovenian government, how satisfied are you with the way it is doing its job?” with responses ranging from (0) extremely dissatisfied to (10) extremely satisfied. Conspiracy theory endorsement was inspected with three indicators, all measured with a 5-point Likert scale (1 = strongly agree; 5 = strongly disagree). The first conspiracy theory was “A small secret group of people is responsible for making all major decisions in world politics”, the second one was related to scientists “Groups of scientists manipulate, fabricate, or suppress evidence in order to deceive the public”, and the third one was COVID-19-associated “Coronavirus is the result of deliberate and concealed efforts of some government or organization”. Conspiracy theories items were reverse coded so that a higher value represents greater agreement.

#### 2.2.2. Sociodemographic Variables

Sociodemographic variables were included, amongst them age (in years), gender (1 = male, 2 = female), and attained educational level (1 = unfinished elementary school; 12 = PhD). Educational levels were recoded into four categories (1 = elementary or less, 2 = secondary, 3 = tertiary (BA or similar), 4 = tertiary (MA, PhD)). Political orientation was tapped with the following question: “In politics people sometimes talk of “left” and “right”. Using this card, where would you place yourself on this scale, where 0 means the left and 10 means the right?”. Political orientation was recoded into five categories (0 = extreme left (1), 1–4 = left-leaning (2), 5 = centrist (3), 6–9 = right-leaning (4), 10 = extreme right (5)).

#### 2.2.3. Outcome Variables

Trust in the government to manage the impact of the COVID-19 pandemic was examined with the question, “Please tell me to what extent you trust the national government in Slovenia to deal with the impact of the coronavirus pandemic. Please answer on a score of 0–10, where 0 means you do not trust the government at all and 10 means you have complete trust.” Satisfaction with the government’s handling of the pandemic was also measured on an 11-point scale (0 = extremely dissatisfied; 10 = extremely satisfied). The item was measured by the question, “Overall, how satisfied are you with the Slovenian government’s handling of the coronavirus pandemic? Please answer this question using this card, where 0 means extremely dissatisfied and 10 means extremely satisfied”. Opinion about following government rules vs. one’s own decisions was asked with the question, “Is it more important for you personally to follow government rules or to make your own decisions when fighting a pandemic? Please answer on a score from 0 to 10 using this card, where 0 means it is much more important to follow government rules and 10 means it is much more important to make your own decisions.”

COVID-19 vaccination intention was measured with the question “Will you get vaccinated against coronavirus with a vaccine that was approved by the national regulatory authority in Slovenia?” with possible answers (1) “Yes, I will”, (2) “Yes, I already have”, and (3) “No”.

### 2.3. Statistical Analyses

Mplus 8 was used for latent profile analysis. Latent profile analysis assumes that individuals can be grouped into profiles (subsamples) with different levels of probability [25], and the essence of the analysis is that it focuses on the individual rather than the variables (i.e., person-oriented approach) [32]. More specifically, the goal of LPA is to identify latent profiles that explain how the observed variables relate to one another. Until the best model fit is established, this is done by adding profiles, with the model parameters being the probability of persons belonging to each profile and the probabilities of belonging to a particular profile. Based on the profile model, the posterior probabilities are provided for each individual [33].

Latent variables used for profile identification were trust in scientists, satisfaction with the Slovenian government, and belief in three different conspiracy theories. The best fitting model was determined based on several statistical information criteria (IC): Akaike information criterion (AIC), Bayesian information criterion (BIC), and sample size adjusted Bayesian information criterion (SABIC). Lower values of IC indicate a better fit. Bootstrap likelihood ratio (BLRT) and Lo–Mendell–Rubin likelihood ratio (LMR) test were used to compare the k_0_ model with the k_−1_ model. A significant value of the BLRT and LMR test shows that the k_0_ solution is superior to the k_−1_ solution. Entropy was also considered, with higher values indicating greater discrimination between profiles [34]. In deciding the best profile solution, we focused particularly on the values of BIC, SABIC, BLRT, and LMR, as entropy and AIC previously selected the number of profiles unsatisfactorily [35].

After deciding on the number of profiles, we proceeded with the analysis in SPSS 26. We used profile probabilities and analyzed whether the profiles differed quantitatively with respect to the five latent variables. We then calculated descriptive statistics to examine the sociodemographic characteristics of each profile. ANOVA was used for analyzing different perceptions on issues about government and the COVID-19 pandemic between profiles. Finally, using multinomial logistic regression, we tested whether the profiles differed in their attitudes toward COVID-19 vaccination.

## 3. Results

### 3.1. Latent Profiles of Trust and Conspiracy Theories Endorsement

Solutions with two to six profiles were compared for profile identification (Table 1). Information criteria (AIC, BIC and SABIC) decreased for all six profiles, with the smallest decrease for the solution going from two to three and five to six profiles. The BLRT was significant for all profile solutions; however, the LMR showed that a three-profile solution fitted the data well. Based on the fit statistics and interpretability of the profiles, we determined that a three-profile solution was the best fit for the data.

ANOVA was used to compare the three profiles based on their characteristics (see Table 2). All three profiles significantly differ regarding trust in scientists, satisfaction with the Slovenian government, and endorsement of conspiracy theories. Regarding trust in scientists, the Hochberg post hoc test (see Table S2, Supplementary Materials) showed that all three profiles significantly differ. Profile 3 showed the most trust in scientists, while Profile 2 expressed the least trust. Satisfaction with government yielded similar results—Profile 3 was the most satisfied, while Profile 2 was the least satisfied with the Slovenian government. Due to the non-homogeneity of variances, the Games–Howell post hoc test was used to examine the differences between profiles for all three conspiracy theories. For all three conspiracy theories, Profile 1 was most likely to endorse them, compared to Profiles 2 and 3. Profile 2 showed greater belief in conspiracy theories compared to Profile 3, which was the least conspiratorial.

**Figure 1:**
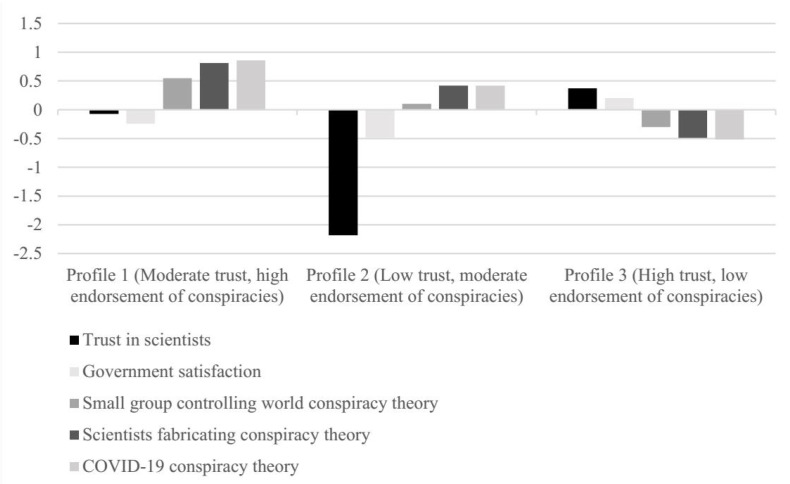
Z-scores of profiles on latent indicator variables.

The profiles shown in Figure 1 were named based on their average values on key variables. The first profile, “Moderate trust, high endorsement of conspiracies” (*n* = 394, 31.4%), consists of individuals with moderate trust in scientists and satisfaction with the government and high endorsement of all three conspiracy theories—the highest endorsement was observed for the belief that COVID-19 was the result of deliberate and concealed efforts of some government or organization. It is also worth mentioning that the group expressed moderate trust in scientists while at the same time expressed belief in conspiracy relating to scientists manipulating, fabricating, or suppressing evidence to deceive the public. Profile 2, “Low trust, moderate endorsement of conspiracies” (n = 114, 9.2%), expressed both the lowest trust in scientists and the lowest satisfaction with the Slovenian government and moderate endorsement of conspiracies. The latter was lower compared to the first profile, but both profiles expressed the most doubt about the origin of COVID-19. The third profile, “High trust, low endorsement of conspiracies” (n = 744, 59.4%), expressed the highest trust in scientists and had the highest satisfaction with government (although the latter was still beneath the midpoint) and expressed the lowest beliefs in conspiracies.

In addition, we were interested in the sociodemographic characteristics of the profiles, which are shown in Table 3. There are no substantial differences between the profiles in terms of age. Regarding gender, Profile 1 (Moderate trust, high endorsement of conspiracies) is evenly represented, while there are more women than men in Profiles 2 and 3. Most individuals in Profiles 1 and 3 have lower levels of education (elementary and high school diplomas), while in Profile 3, individuals mainly have a high school or university degree. All three profiles are predominantly composed of individuals who lean toward the political center and individuals who lean toward the left. Comparing the profiles, we find that extreme left-wing individuals are over-represented in Profile 2, right-leaning individuals are primarily found in Profile 3, and individuals who identify with extreme right-wing political views are found in Profiles 1 and 3.

### 3.2. Government and COVID-19 Pandemic

We also examined whether profiles differed in terms of their trust in the government to handle the consequences of the COVID-19 pandemic, whether they were satisfied with how the government was handling the pandemic, and whether there were differences in their perceptions of following government regulations or making their own decisions regarding the pandemic. ANOVA (see Table 4) confirmed that the profiles significantly differed on all three observed variables. The Games–Howell post hoc test (see Table S3, Supplementary Materials) confirmed that Profile 3 was significantly more trusting and satisfied with how the government was handling the pandemic, and Profile 2 was least trusting. However, there were no significant differences between Profile 1 and Profile 2 in terms of satisfaction with the government and the pandemic. Regarding following rules, Hochberg’s post hoc test revealed that Profiles 1 and 2 appeared to be more individualistic (i.e., they placed more importance on following their own decisions) than Profile 3. However, Profiles 1 and 2 did not statistically differ on this variable.

### 3.3. Differences between the Latent Profiles and Their Attitudes toward COVID-19 Vaccination

**Figure 2:**
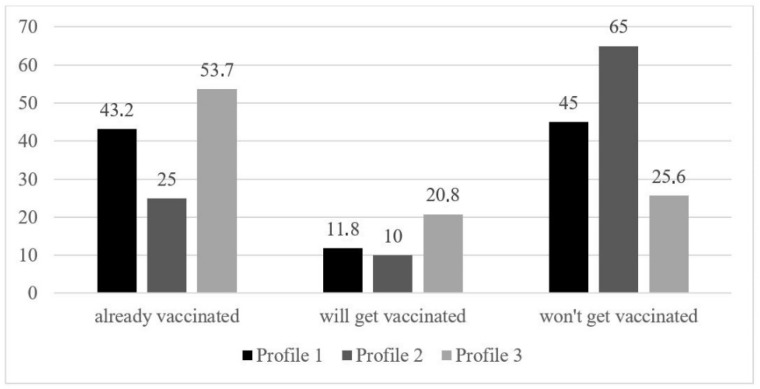
COVID-19 vaccination intention across latent profiles (%).

Finally, we examined whether latent profiles differed in their attitudes toward vaccination against COVID-19. Figure 2 shows the percentages of pro-vaccination decisions based on profile membership. Profile 3 (High trust, low endorsement of conspiracies) showed the most positive attitudes toward vaccination. Individuals in Profile 3 were most likely to have already been vaccinated (53.7%), followed by Profile 1 (Moderate trust, high endorsement of conspiracies) (43.2%), which indicates a ten-percentage point difference in vaccine uptake. Profile 2 (Low trust, moderate endorsement of conspiracies) was least likely to get vaccinated (65% answered they won’t get vaccinated). Summing past and intended future vaccine uptake showed similar differences, with 75% of Profile 3 being pro-vaccine, 55% of Profile 1 and only 35% among Profile 2. We further examined the likelihood of vaccination acceptance among profiles. Multinomial regression (see Table 5) revealed that both Profile 1 (β = −0.78) and Profile 2 (β = −1.70) were less likely to have already been vaccinated or get vaccinated, compared with Profile 3 (Profile 1: β = −1.13; Profile 2: β = −1.66). Based on the beta coefficients, Profile 2 was the least likely of all profiles to have been or would get vaccinated against COVID-19.

## 4. Discussion

Based on latent profile analysis, we identified three distinct profiles that differ in their trust in scientists, satisfaction with the Slovenian government, and belief in conspiracy theories. The profiles also differ in their intention to be vaccinated against COVID-19 and in certain beliefs about the government’s role in preventing and managing a pandemic.

The first profile (Moderate trust, high endorsement of conspiracies) had a moderate level of trust in scientists and a slightly lower level of satisfaction with the government. In contrast, it had the highest score for belief in conspiracy theories among the three profiles. Interestingly, of the three conspiracy theories, the COVID-19 conspiracy theory was the one most believed in among group members. The second profile (Low trust, moderate endorsement of conspiracies) showed the least trust in scientists and the least satisfaction with government, but a moderate belief in conspiracy theories. The third profile (High trust, low endorsement of conspiracies) had the highest trust in scientists, was most satisfied with the government, and had low belief in conspiracy theories.

We also found that the third profile was most satisfied with and trusted the government’s work on the COVID-19 pandemic and its aftermath. This profile also showed the greatest willingness to follow the government’s instructions regarding the pandemic. The second profile was the least trusting and satisfied and believed it was necessary to follow one’s own decisions rather than the government’s instructions.

In addition, Profile 3 was the most positive about vaccination, showing the greatest willingness to be vaccinated or indicating that they had already received the vaccine. We consider Profile 1 as vaccine-hesitant because they were less likely to be vaccinated. In contrast, individuals in Profile 2 were opposed to vaccination because they were less likely to be vaccinated and, at the same time, they were most likely to express the view that they did not want to be vaccinated.

Our results are consistent with previous studies showing dissatisfaction with government [21,22] and distrust in scientists [6,8,14,15,16] are associated with greater vaccine hesitancy or rejection of vaccination. There is also converging evidence that conspiracy theories negatively predict vaccination [7,13]. Our study also confirmed that conspiracy endorsements are mutually reinforcing, even if they differ in content, thus representing a monologic belief system [36,37]. In other words, both Profile 1 and 2, who expressed conspiratorial thinking to some extent, believed in all three observed conspiracies, while individuals from Profile 3 distrusted all three.

The study illustrates the importance of attitudes toward government and science in vaccination attitudes. In particular, Profile 3 (Hight trust, low endorsement of conspiracies) was most consistent with previous studies, as trust in science, satisfaction with government, and dislike of conspiracy theories were associated with higher vaccination intentions. On the other hand, significant differences were found when observing Profile 1 (Moderate trust, high endorsement of conspiracies) and Profile 2 (Low trust, moderate endorsement of conspiracies). Profile 2 was shown to be even more vaccine-rejecting than Profile 1, which was otherwise the most conspiratorial of the three. Even though both profiles sympathized with conspiracy theories to some extent, they significantly differed in their attitudes toward government and their trust in science. Our results indicate that the degree of trust in scientists and satisfaction with the government in the presence of conspiracy theories is vital for vaccination decisions. More specifically, distrust, dissatisfaction, and the presence of conspiracy theories are the “perfect storm” for vaccination rejection. In contrast, despite conspiracy theories, a certain level of trust and satisfaction may reduce vaccination rejection. Therefore, Profile 1, despite being more conspiratorial, included a higher percentage of already vaccinated individuals and was slightly less rejecting compared to Profile 2. The study, thus, highlights another aspect of the importance of trust and satisfaction with key institutions in achieving higher vaccination rates.

Based on the results, some practical recommendations can be drawn. Most importantly, the communication and transmission of information should be adapted to the characteristics of the groups. We found that the vaccine-hesitant group endorsed conspiracy theories but simultaneously trusted scientists to some extent and expressed some satisfaction with the government. This means that scientists and government institutions can access this group through communication. It may be helpful to communicate to the public the facts about vaccination as effectively as possible and try to dispel misinformation and conspiracy theories. With transparent and evidence-based information, this group’s confidence in vaccination could increase.

On the other hand, the minority that opposed vaccination also distrusted scientists and was dissatisfied with the government. This group was also much less likely to follow government instructions. This means vaccine-related advice from official institutions and scientists will likely not be heeded. In interpersonal interactions between vaccination supporters and opponents, the suggestion of scientific evidence can be even more polarizing [38]. Rather than providing facts (i.e., communicating scientific evidence), which this group is less likely to trust, one critical goal is identifying and addressing the group’s concerns. The present study did not examine the reasons for distrust in science and dissatisfaction with the government. Therefore, future studies and campaigns must address this critical question as well.

The study also has some limitations. The data are cross-sectional, making it impossible to infer cause and effect. Secondly, the sample was predominantly distrustful of Slovenian politics and government; therefore, all profiles were below the mid-point when examining satisfaction with government. Thirdly, the relationship between the observed variables and willingness to be vaccinated against COVID-19 could be caused by other factors, as the confounding effect of numerous potential factors has not been excluded. Future studies should further examine the interaction between institutional trust and conspiracies, as well as other potential determinants of vaccine hesitancy.

## 5. Conclusions

Slovenians, who are less trusting of scientists, more dissatisfied with the Slovenian government, and at the same time endorse conspiracy theories to some extent, are less likely to be vaccinated against COVID-19. On the other hand, the group of individuals more inclined to vaccinate is more trusting of scientists, more satisfied with the government, and less likely to endorse conspiracies. Policymakers should consider different subgroups of individuals and their characteristics and attitudes towards the COVID-19 vaccine when tackling vaccine hesitancy and communicating about COVID-19.

## Figures and Tables

**Table 1:** Fit indices for profile solutions.

Profile	LL	AIC	BIC	SABIC	Entropy	BLRT-p	LMR-p	Smallest Class %
1	−11966.490	23952.981	24004.306	23972.541	/	/	/	/
2	−11731.906	23495.812	23577.932	23527.109	0.60	<0.001	<0.001	36.2
**3**	**−11664.573**	**23373.146**	**23486.061**	**23416.179**	**0.67**	**<0.001**	**0.0196**	**9.1**
4	−11615.361	23286.721	23430.431	23341.490	0.70	<0.001	0.1532	4.8
5	−11558.414	23184.827	23359.332	23251.333	0.82	<0.001	0.0016	2.9
6	−11529.057	23138.113	23343.413	23216.355	0.84	<0.001	0.1868	2.0

Note. LL = Log likelihood value; AIC = Akaike’s information criterion, BIC = Bayesian information criterion; SABIC = sample size adjusted Bayesian information criterion; BLRT-p = *p*-value of the bootstrap likelihood ratio test; LMR-p = Lo–Mendell–Rubin likelihood ratio test. BLRT and LMR are tested for significance, while AIC, BIC, and SABIC values are compared across different profile solutions [34]. The model that shows the best data fit is marked in bold.

**Table 2:** Differences of profiles on latent variables (ANOVA).

	Profile 1 (31.4%) (a)		Profile 2 (9.2%) (b)		Profile 3 (59.4%) (c)		F	η^2^
	M	SD	M	SD	M	SD		
Trust in scientists	6.99 ^b,c^	1.56	2.42 ^c^	1.36	7.96	1.49	674.737 ***	0.52
Satisfaction with government	3.08 ^b,c^	2.79	2.36 ^c^	2.73	4.35	2.84	40.997 ***	0.06
Small group controlling world conspiracy theory	4.11^b,c^	1.11	3.57 ^c^	1.32	3.07	1.11	113.601 ***	0.15
Scientists fabricating conspiracy theory	4.18 ^b,c^	0.96	3.71 ^c^	1.17	2.59	0.95	364.604 ***	0.36
COVID-19 conspiracy theory	4.38 ^b,c^	1.10	3.78 ^c^	1.20	2.50	0.98	421.600 ***	0.41

Notes. *** *p* < 0.001. Trust in scientists and satisfaction with the government were measured on an 11-point scale. Conspiracy theories were measured with a 5-point Likert scale. Due to the non-homogeneity of variances, Welch’s F is reported for all three indicators of conspiracy theories endorsement. Letters in superscripts indicate significant pairwise comparisons between profiles based on post hoc tests.

**Table 3:** Sociodemographic characteristics of profiles.

		Profile 1		Profile 2		Profile 3	
		M	SD	M	SD	M	SD
	Age	51.12	19.4	47.89	15.36	48.73	19.22
		*f*	%	*f*	%	*f*	%
Gender	Male	197	50	52	45.6	342	46
	Female	197	50	62	54.4	402	54
Education	Elementary or <	75	19.3	27	23.7	104	14
	Secondary	236	60.7	67	58.8	363	48.9
	Tertiary (BA or similar)	67	17.2	19	16.7	229	30.8
	Tertiary (MA, PhD)	11	2.8	1	0.9	47	6.3
Political orientation	Extreme left	28	9	10	11.5	40	5.9
	Left-leaning	70	22.5	17	19.5	147	21.6
	Centrist	139	44.7	42	48.3	305	44.8
	Right-leaning	55	17.7	14	16.1	143	21
	Extreme right	19	6.1	4	4.6	46	6.8

**Table 4:** Differences between profiles regarding the COVID-19 pandemic and government.

	Profile 1 (a)		Profile 2 (b)		Profile 3 (c)		F	η^2^
	M	SD	M	SD	M	SD		
Satisfaction with government’s dealing with pandemic	4.13 ^c^	2.88	3.41 ^c^	3.06	5.15	2.59	28.301 ***	0.04
Trusting government dealing with pandemic’s impact	4.06 ^b,c^	2.87	2.93 ^c^	2.88	5.13	2.63	40.253 ***	0.07
Following government rules vs. own decisions when fighting pandemic	5.78 ^c^	3.16	6.39 ^c^	3.25	4.87	3.04	18.716 ***	0.03

Notes. *** *p* < 0.001. Due to the non-homogeneity of variances, Welch’s F is reported for the second and third variable. Letters in superscripts indicate significant pairwise comparisons between profiles based on post hoc tests.

**Table 5:** Multinomial regression of COVID-19 vaccination intention.

	Profile 1 “Moderate Trust, High Endorsement of Conspiracies”		Profile 2 “Low Trust, Moderate Endorsement of Conspiracies”	
	β (SE)	Wald	β (SE)	Wald
already have	−0.78 (0.21) ***	13.504	−1.70 (0.33) ***	26.014
will have ^a^	−1.13 (0.30) ***	13.960	−1.66 (0.47) ***	12.577

Note. ^a^ reference category is “won’t get vaccinated”. Profile 3 (High trust, low endorsement of conspiracies) is the reference. CTE = conspiracy theories endorsement. *** *p* < 0.001.

## Data Availability

The data presented in this study are available at https://ess-search.nsd.no/en/study/172ac431-2a06-41df-9dab-c1fd8f3877e7 (accessed on 25 July 2022).

## Supplementary Materials

The following supporting information can be downloaded at: https://www.mdpi.com/article/10.3390/ijerph191610096/s1, Table S1: Descriptive statistics of key variables; Table S2: Post hoc test of profile differences on latent variables; Table S3: Post hoc test of differences between profiles regarding the COVID-19 pandemic and government.
