# Supplementary material for: Distrustful, Dissatisfied, and Conspiratorial: A Latent Profile Analysis of COVID-19 Vaccination Rejection
Source: Int J Environ Res Public Health. 2022 Aug 15;19(16):10096. doi: 10.3390/ijerph191610096 (PMC9408605; doi:10.3390/ijerph191610096)
Supplement: Supplementary file 1 [file ijerph-19-10096-s001.zip › ijerph-1860064-supplementary-final.pdf]

Table S1. Descriptive statistics of key variables.

	M	SD	1	2	3	4	5	6	7	8	9	10	11	12	13	14	15
1. Trust in parliament	3.86	2.42	-														
2. Trust in politicians	2.72	2.25	.69**	-													
3. Trust in political parties	2.78	2.22	.64**	.83**	-												
4. Trust in scientists	7.15	2.17	.37**	.28**	.27**	-											
5. Satisfaction with government	3.77	2.90	.53**	.59**	.52**	.13**	-										
6. Small group controlling the world conspiracy theory	3.44	1.22	-.10**	-.07*	-.08**	-.09**	-0.01	-									
7. Scientists fabricating conspiracy theory	3.19	1.22	-.18**	-.15**	-.14**	-.24**	-.08**	.37**	-								
8. COVID-19 conspiracy theory	3.21	1.36	-.22**	-.18**	-.18**	-.25**	-.17**	.25**	.41**	-							
9. Satisfaction with government's dealing with pandemic	4.68	2.79	.38**	.41**	.36**	.15**	.63**	-0.05	-.10**	-.19**	-						
10. Trusting government dealing with pandemic	4.60	2.82	.45**	.47**	.43**	.18**	.71**	-0.06	-.10**	-.20**	.77**	-					
11. Following government or own rules when fighting the pandemic	5.30	3.14	-.26**	-.23**	-.21**	-.14**	-.33**	0.03	.09**	.17**	-.39**	-.39**	-				
12. COVID-19 vaccination	2.19	0.70	-.19**	-.16**	-.15**	-.23**	-.23**	0.05	.20**	.23**	-.26**	-.27**	.30**	-			
13. Age	49.41	18.99	-0.04	-.04	-0.04	0.04	.07*	0.04	.14**	0.01	.27**	.22**	-.06*	-.13**	-		
14. Gender	1.53	0.50	-0.03	0.02	-0.00	-0.04	-0.05	-0.04	-0.05	0.01	0.01	-0.01	0	0.08	0.02	-	
15. Education	2.18	0.76	.11**	0.02	0.02	.19**	-.07*	-0.02	-.16**	-.18**	-.07*	-.06*	0.05	-0.07	-.08**	.08**	-
16. Political orientation	2.96	0.98	.23**	.26**	.22**	-0.02	.54**	-0.01	-0.02	0.01	.41**	.45**	-.20**	-.11*	-0.00	0.01	-.15**

Note. ** $p < 0.01$, * $p < 0.05$.

Table S2. Post hoc test of profile differences on latent variables.

		Group comparison	Mean difference	SE
Hochberg's post hoc test	Trust in scientists	Profile 1 vs. Profile 2	4.57***	0.16
		Profile 1 vs. Profile 3	-0.97***	0.09
		Profile 2 vs. Profile 3	-5.54***	0.15
	Satisfaction with government	Profile 1 vs. Profile 2	0.72*	0.3
		Profile 1 vs. Profile 3	-1.27***	0.18
		Profile 2 vs. Profile 3	-1.99***	0.28
Games Howell's post hoc test	The small group leads the world	Profile 1 vs. Profile 2	0.54***	0.14
		Profile 1 vs. Profile 3	1.04***	0.07
		Profile 2 vs. Profile 3	0.50***	0.13
	Scientists fabricating	Profile 1 vs. Profile 2	0.45***	0.12
		Profile 1 vs. Profile 3	1.58***	0.06
		Profile 2 vs. Profile 3	1.12***	0.11
	COVID-19 conspiracy	Profile 1 vs. Profile 2	0.60***	0.13
		Profile 1 vs. Profile 3	1.87***	0.07
		Profile 2 vs. Profile 3	1.28***	0.12

Note. *** $p < 0.001$, * $p < 0.05$.

Table S3. Post hoc test of differences between profiles regarding the COVID-19 pandemic and government.

		Group comparison	Mean difference	SE
Hochberg's post hoc test	Following the government's or own rules when fighting the pandemic	Profile 1 vs. Profile 2	-0.60	0.33
		Profile 1 vs. Profile 3	0.91***	0.19
		Profile 2 vs. Profile 3	1.51***	0.31
Games Howell's post hoc test	Satisfaction with government	Profile 1 vs. Profile 2	0.72	0.32
		Profile 1 vs. Profile 3	-1.02***	0.18
		Profile 2 vs. Profile 3	-1.74***	0.3
	Trusting government dealing with pandemic	Profile 1 vs. Profile 2	1.13***	0.31
		Profile 1 vs. Profile 3	-1.07***	0.18
		Profile 2 vs. Profile 3	-2.20***	0.29

Note. *** $p < 0.001$.
